# Experimental Demonstration of a Synthetic Lorentz Force by Using Radiation Pressure

**DOI:** 10.1038/srep13485

**Published:** 2015-09-02

**Authors:** N. Šantić, T. Dubček, D. Aumiler, H. Buljan, T. Ban

**Affiliations:** 1 Department of Physics, University of Zagreb, Bijenička c. 32, 10000 Zagreb, Croatia; 2 Institute of Physics, Bijenička c. 46, 10000 Zagreb, Croatia

## Abstract

Synthetic magnetism in cold atomic gases opened the doors to many exciting novel physical systems and phenomena. Ubiquitous are the methods used for the creation of synthetic magnetic fields. They include rapidly rotating Bose-Einstein condensates employing the analogy between the Coriolis and the Lorentz force, and laser-atom interactions employing the analogy between the Berry phase and the Aharonov-Bohm phase. Interestingly, radiation pressure - being one of the most common forces induced by light - has not yet been used for synthetic magnetism. We experimentally demonstrate a synthetic Lorentz force, based on the radiation pressure and the Doppler effect, by observing the centre-of-mass motion of a cold atomic cloud. The force is perpendicular to the velocity of the cold atomic cloud, and zero for the cloud at rest. Our novel concept is straightforward to implement in a large volume, for a broad range of velocities, and can be extended to different geometries.

Experiments on synthetic magnetic/gauge fields for neutral atoms^1^,^2^,^3^,^4^,^5^,^6^,^7^,^8^,^9^,^10^,^11^ have enabled realizations of the Hall effect^4^, famous Hamiltonians such as the Harper^7^,^8^ and the Haldane Hamiltonian^10^, intriguing topological effects^9^,^10^, and the observation of synthetic Dirac monopoles^11^. There are a few recent reviews on this promising theoretical and experimental progress in synthetic magnetic/gauge fields^12^,^13^,^14^,^15^. The first implementation of synthetic magnetism was in rapidly rotating Bose-Einstein condensates (BECs), employing the analogy between the Lorentz force and the Coriolis force^1^,^2^. The methods based on laser-atom interaction employ the analogy between the Berry phase in atomic systems^16^, and the Aharonov-Bohm phase for charged particles^13^. The first of them was realized in the NIST group with spatially dependent Raman optical coupling between internal hyperfine atomic states in bulk BECs^3^. Methods generating synthetic magnetic fields in optical lattices engineer the complex tunnelling matrix elements between lattice sites^5^,^6^,^7^,^8^,^9^. They include shaking of the optical lattice^6^, laser assisted tunnelling in optical superlattices realizing staggered synthetic magnetic fields^5^, in tilted lattices realizing homogeneous fields^7^,^8^, and an all-optical scheme which enables flux rectification in optical superlattices^9^. Interestingly, radiation pressure has not yet been used among the methods for synthetic magnetism, that is, to create the analogue of the Lorentz force. Here we experimentally demonstrate the synthetic Lorentz force based on radiation pressure in cold atomic gases. We measure the dependence of the transverse radiation pressure force (analogous to the transverse Hall deflection) on the velocity of a cold atomic cloud by observing the centre-of-mass (CM) motion. The observed force is perpendicular to the velocity, and zero for the atomic cloud at rest. This concept based on radiation pressure, theoretically proposed in Ref. [17], is straightforward to implement in a large volume (e.g., volumes 1 mm^3^–1 cm^3^ are easily accessible)^18^, is applicable for a broad range of velocities, and can be extended to different geometries. The main reason for the absence of radiation pressure from the previously used methods of synthetic magnetism is the associated heating due to spontaneous emission. However, this is not an obstacle for atomic gases cooled and trapped in a Magneto-Optical Trap (MOT), where our experiments are performed.

## Results

### The basic idea

**Figure 1:**
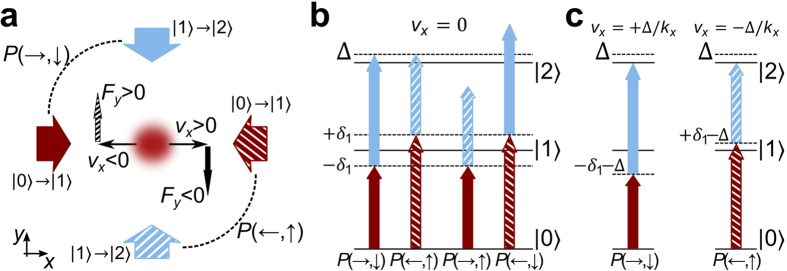
The scheme used to obtain the synthetic Lorentz force via radiation pressure. It is based on two-step two-photon transitions involving three atomic levels. (**a**) Two counter-propagating laser beams on the *x*-axis (red arrows) drive the 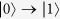 transition, while the 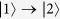 transition is driven by two counterpropagating beams on the *y*-axis (blue arrows). The transverse radiation pressure force arising from the 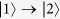 transition *F*_*y*_, depends on the velocity *v*_*x*_ as indicated. (**b**) Four possible excitation pathways and a sketch of the detuning values: *P*(→, ↓) denotes absorption of a 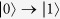 photon going towards the positive *x*-direction, followed by absorption of a 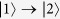 photon travelling towards the negative *y*-direction, and so on. The total detuning value for *P*(→, ↓) and *P*(←, ↑) is Δ, whereas it is much larger in magnitude for *P*(→, ↑) and *P*(←, ↓); the two latter pathways are thus negligible in this configuration. (**c**) The Doppler shift provides *F*_*y*_ as a function of *v*_*x*_ as sketched in (**a**): pathway *P*(→, ↓) for an atom with positive velocity *v*_*x*_ = +Δ/*k*_*x*_ (*v*_*y*_ = 0) is on resonance, providing negative *F*_*y*_. Likewise, *P*(←, ↑) is on resonance for an atom with negative velocity *v*_*x*_ = −Δ/*k*_*x*_, providing positive *F*_*y*_. See text for details.

The idea behind our experiment is to drive two-step two-photon transitions between three atomic levels, 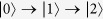, with mutually perpendicular laser beams as illustrated in Fig. 1. Two counterpropagating laser beams aligned with the *x*-axis drive the 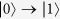 transition, whereas the 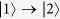 transition is driven by counterpropagating beams aligned on the *y*-axis. Due to the Doppler effect and the perpendicular configuration of the laser beams, both components of the radiation pressure force depend on both components of the atomic velocity: 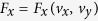 and 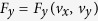. This gives us the opportunity to design the detuning values of our lasers such that *F*_*y*_ is positive/negative for atoms with negative/positive velocity component *v*_*x*_, and that the total force is zero for an atom at rest: 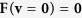. These are the characteristics of the synthetic Lorentz force that we experimentally demonstrate.

The design of the detuning values of the lasers is crucial in obtaining the desired result. The beams driving the first step of the transition 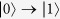 are detuned by the same magnitude, but with the opposite sign. The one towards the positive *x*-direction is red-detuned by *δ*_→_ 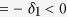, while the other is blue-detuned by *δ*_←_ 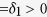. Their intensities are equal. Thus, if just these two lasers were present, the net force on atoms (of any velocity) would be zero. However, the population of level 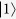 would depend on the velocity *v*_*x*_, which implies that the rate of transitions 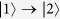 giving the transverse force will depend on *v*_*x*_. The detuning values of the beams driving the second step of the transition, 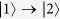 are denoted by *δ*_↑_ and *δ*_↓_, for the beam propagating in the positive and negative *y* direction, respectively. For now, let us set these values such that *δ*_→_ + *δ*_↓_ = *δ*_←_ + *δ*_↑_ = Δ > 0, as indicated in Fig. 1(b).

The two-step two-photon transitions, where absorption of a 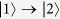 photon follows absorption of a 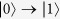 photon with perpendicular momentum, yield the synthetic Lorentz force via momentum transfer from photons to atoms. Given the fact that we have two counterpropagating beams for each transition, we have four excitation pathways for the two-step two-photon transition, denoted by *P*(→, ↑), *P*(←, ↑), *P*(→, ↓), and *P*(←, ↓), see Fig. 1. The arrows correspond to the direction of the photon’s momentum, for example, *P*(→, ↓) denotes the pathway where absorption of a photon travelling in the +*x* direction is followed by absorption of a photon in the −*y* direction and so on. Since the detuning magnitudes of the first step are identical for all pathways (|*δ*_→_| = *δ*_←_), the relevant quantity is the total detuning for the two-step two-photon transitions. It is important to note that the total detuning Δ for pathways *P*(→, ↓) and *P*(←, ↑), is much smaller in magnitude than the detuning values of *P*(→, ↑) and *P*(←, ↓). The last two are thus negligible in the setup of Fig. 1.

To understand the origin of the synthetic Lorentz force, we take into account the Doppler shift. For an atom moving along the *x*-axis with velocity *v*_*x*_ (*v*_*y*_ = 0), the *P*(←, ↑) pathway is detuned by Δ + *k*_*x*_*v*_*x*_, i.e., it is on resonance when *v*_*x*_ = −Δ/*k*_*x*_ < 0. Because photons from the second step of *P*(←, ↑) impart momentum towards the positive *y*-direction, there will be a positive force *F*_*y*_ for atoms with negative *v*_*x*_. In the same fashion, the *P*(→, ↓) pathway will be on resonance when *v*_*x*_ = +Δ/*k*_*x*_ > 0 yielding negative *F*_*y*_ for positive *v*_*x*_.

### The experiment

In the experiment, we use cold ^87^Rb atoms. For the 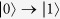 transition we use the D2 transition in ^87^Rb: 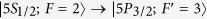 at 780 nm^19^. The easily accessible transition, 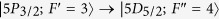 at 776 nm^20^, is used for the second step 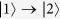. The linewidths of the states are 2*π* × 6.1 MHz for the 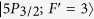 state^19^, and 2*π* × 0.66 MHz for the 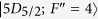 state^20^.

The ^87^Rb atoms are cooled and trapped in a standard glass vapour cell magneto-optical trap (MOT), arranged in a retro-reflected configuration. In this configuration, three orthogonal retro-reflected beams are used to create the total of six beams needed for the MOT. A pair of anti-Helmholtz coils provides a quadrupole magnetic field, which together with the laser beams creates a trapping potential (for example, see Ref. [18]). Fluorescence imaging of the cloud is performed with a camera aligned along the *z*-axis. In typical experimental conditions we obtain a cloud of 0.4 mm in diameter, which contains about 10^8^ atoms of ^87^Rb, at a temperature of 50 *μ*K (for details of the experimental setup see Methods). The four beams implementing the synthetic Lorentz force, arranged as in Fig. 1, are of much smaller intensity than the MOT beams. Therefore, they are negligible when the MOT beams are ON. For the experimental detection of the synthetic Lorentz force, we turn the MOT beams OFF, as described in detail below.

We need to measure the transverse force *F*_*y*_ in dependence of the velocity *v*_*x*_ of the atomic cloud. Thus, we must prepare a cloud with a given centre of mass (CM) velocity. For this purpose we use an additional pair of current coils that produce a bias magnetic field along the symmetry axis of the anti-Helmholtz coils, *x* in our notation here. The bias field moves the centre of the trap (the point where **B** = 0), which displaces the cloud approximately 1 mm along the *x*-axis.

The measurement protocol is as follows. (i) We load the trap with the bias magnetic field on. (ii) At *t* = −2 ms we reverse the bias magnetic field by reversing the current, which suddenly shifts the centre of the trap. This introduces a force on the cloud due to the MOT beams. During the next 2 ms the cloud accelerates along the *x*-axis towards the new trap centre. (iii) Next we turn off the MOT cooling laser and all real magnetic fields. This moment corresponds to *t* = 0 in our presentation. The system is now simplified because Zeeman splitting of hyperfine levels is absent and the radiation force left, arising solely from the lasers implementing the synthetic Lorentz force, is not spatially dependent (it is only velocity-dependent). (iv) The cloud starts to expand because the trapping is absent, but it also moves in the *xy* plane due to both the initial velocity, *v*_*x*_(*t* = 0), and the radiation pressure force. (v) After some delay time *t*, the cooling laser is suddenly turned on and the cloud is imaged with the camera. From the trajectory traversed by the CM of the cloud (*x*(*t*), *y*(*t*)), we can find the force acting on the atoms. If we wish to image a cloud initially at rest we skip steps (i) and (ii). For a given delay time *t*, we repeat the measurement protocol 20 times in identical conditions, and subsequently average to obtain *x*(*t*) and *y*(*t*). The gravity is along the *z*-axis in our system; free fall of atoms due to gravity does not affect the motion in the *xy* plane. It should be stated that we perform these measurements first with 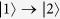 lasers OFF, and then with these lasers ON, keeping all other parameters identical. The difference in the path *y*(*t*), with lasers 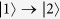 ON and OFF, gives us the transverse motion due to the synthetic Lorentz force.

### The experimental proof of the synthetic Lorentz force by radiation pressure

**Figure 2:**
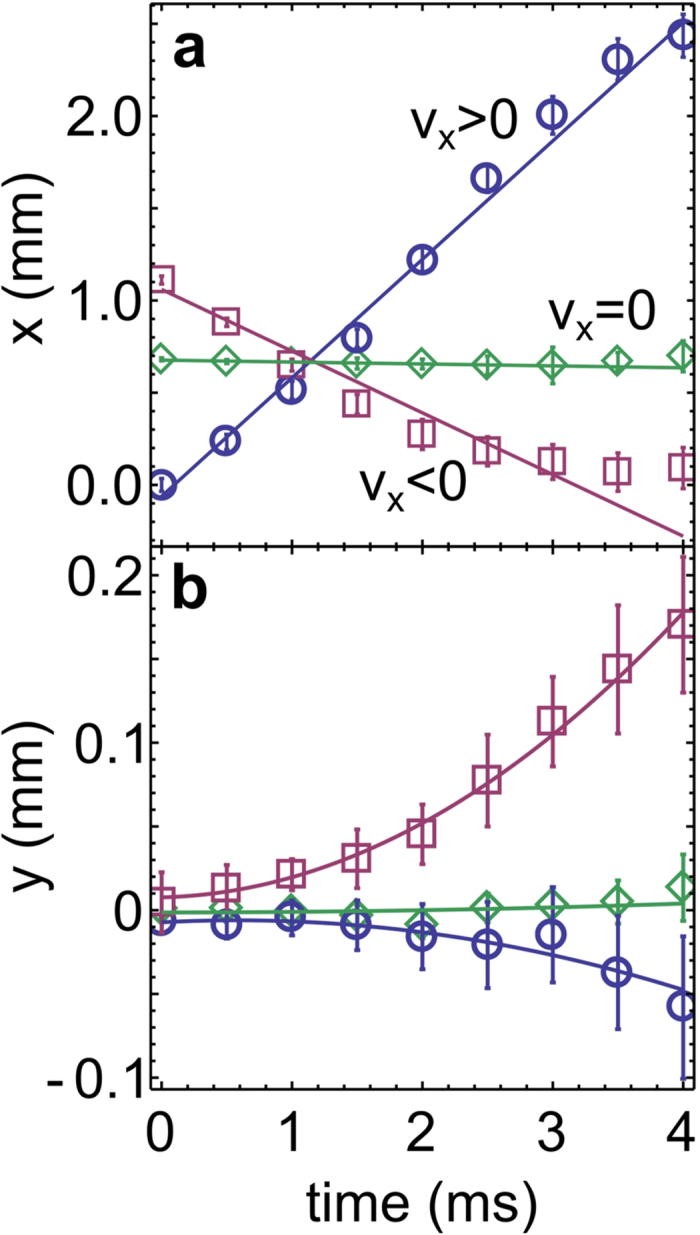
The trajectories of the CM of the atomic cloud in the presence of the synthetic Lorentz force. (**a**) *x*(*t*), and (**b**) *y*(*t*) for three different initial velocities, *v*_*x*_ = 0.6 m/s >0 (circles), *v*_*x*_ = −0.3 m/s <0 (squares), and *v*_*x*_ = 0 m/s (diamonds); initial component of *v*_*y*_ = 0 in all measurements. Accelerating motion along *y* is the signature of the transverse force *F*_*y*_, which depends on *v*_*x*_. The lines are fitted to the experimental data, linear fits for *v*_*x*_(*t*), and quadratic for *v*_*y*_(*t*).

The results of the experiment are illustrated in Fig. 2. We show the trajectory of the cloud (*x*(*t*), *y*(*t*)), in the presence of the synthetic Lorentz force, for three initial velocities: *v*_*x*_ = −0.3 m/s (squares), *v*_*x*_ = 0 m/s (diamonds), and *v*_*x*_ = 0.6 m/s (circles); *v*_*y*_ = 0 at *t* = 0 in each run of the experiment. There is a difference in the magnitude of the initial *v*_*x*_ for the positive and negative velocity [circles and squares in Fig. 2(a)], which is a result of our MOT retro-reflected geometry, and the way we accelerate the cloud in step (ii) of the protocol. In order to prepare a cloud with positive *v*_*x*_, the cloud is accelerated with the incoming MOT beam (coming from the laser side of the setup), whereas acceleration in the opposite direction is performed with the reflected beam which has smaller intensity. The reflected beam intensity is smaller due to the losses, which are a result of the passage of the incoming beam through the dense cloud (absorption), and partially due to reflection. Consequently, the negative initial velocity is smaller than the positive velocity.

For the lasers implementing the synthetic Lorentz force, we use the following detuning values: for the first step at 780 nm *δ*_←_ = −*δ*_→_ = 2*π* × 6 MHz, and for the second step at 776 nm *δ*_↑_ = −2*π* × 3.5 MHz and *δ*_↓_ = 2*π* × 7.1 MHz. The two operational two-step pathways are *P*(←, ↑) and *P*(→, ↓), whereas the other two are far from resonance. The intensities of the beams used are *I*_780_ = 0.060 mW/cm^2^ and *I*_776_ = 2.9 mW/cm^2^, giving Rabi frequencies Ω_780_ = 2*π* × 1.2 MHz and Ω_776_ = 2*π* × 0.94 MHz.

By inspection of Fig. 2, we see that the cloud travels along the *x*-axis by inertia, whereas it accelerates along the *y*-axis due to the synthetic Lorentz force. The direction of the force depends on the sign of the velocity *v*_*x*_ (negative/positive *v*_*x*_ gives positive/negative *F*_*y*_), and the force is zero for a cloud at rest. We observe an asymmetry in the force *F*_*y*_, for the positive and negative velocity. In order to justify the exact choice of the detuning values *δ*_↑_ and *δ*_↓_, and further investigate the observed asimmetry in the measured synthetic Lorentz force, we perform measurements in a slightly simplified configuration.

### Measurements in an auxiliary configuration

**Figure 3:**
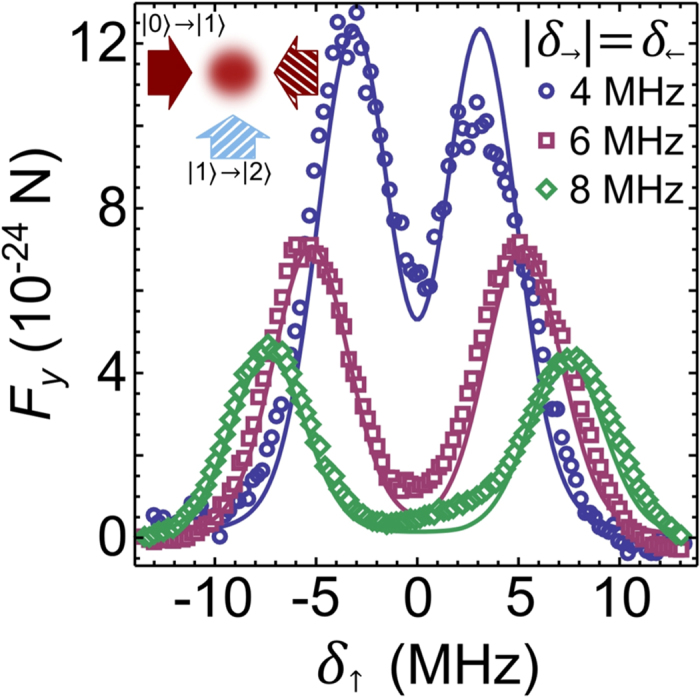
Frequency scan of the two-step two-photon resonance in the auxiliary configuration. The calculated (solid lines) and measured force *F*_*y*_ as a function of *δ*_↑_ for the excitation with just three beams as shown. Measurements are performed for the cloud with initial velocity zero. The plots show resonances for three values of the detuning *δ*_←_ = −*δ*_→_ = 2*π* × 4 MHz (circles), 6 MHz (squares) and 8 MHz (diamonds).

We inspect the force along the *y* direction arising from the two-step two-photon resonances, by using a configuration with three laser beams illustrated in Fig. 3. We block the beam pointing towards the negative *y*-direction, and measure *F*_*y*_ arising from the remaining beam (the positive *y*-direction), which drives the 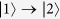 transition. The force is measured for an atomic cloud with velocity zero, as a function of the detuning *δ*_↑_, see Fig. 3. Measurements are done for three different detuning values of the first-step 780 nm beams *δ*_←_ = −*δ*_→_ = 2*π* × 4, 6, 8 MHz. The intensities of the lasers driving the transitions are now *I*_780_ = 0.043 mW/cm^2^ and *I*_776_ = 4.8 mW/cm^2^, giving Rabi frequencies Ω_780_ = 2*π* × 1.1 MHz and Ω_776_ = 2*π* × 1.2 MHz. The two maxima in Fig. 3 are profiles of the two-step two-photon resonances: the peak in the vicinity of *δ*_↑_ = −*δ*_→_ > 0 corresponds to the excitation pathway *P*(→, ↑), and the peak close to *δ*_↑_ = −*δ*_←_ < 0 corresponds to *P*(←, ↑).

Solid lines in Fig. 3 show the theoretically calculated profiles of *F*_*y*_ (see Methods for details of the theoretical calculation). The agreement between theory and experiment is evident. All parameter values used in the theoretical calculation are taken from the experiment, except that the Rabi frequencies are reduced by 20%. This is reasonable because in the experiment, the absorption of laser beams across a dense atomic cloud reduces their intensity^21^.

It should be pointed out that the peaks in *F*_*y*_(*δ*_↑_) are slightly displaced from the values *δ*_↑_ = ±|*δ*_→_|, towards *δ*_↑_ = 0. For example, for |*δ*_→_| at 6 MHz, the maxima are at ±5.3 MHz. Moreover, the FWHM of the peaks is larger than the linewidth of the state 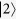. These two observations are a consequence of the laser linewidth, which is larger than the linewidth of the state 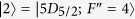 (see Methods). The finite linewidth of 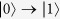 lasers at 780 nm distorts the peaks as follows: the side of the peak closer to *δ*_↑_ = 0 is lifted up in comparison the opposite side of the peak. This follows from the fact that the two-step two-photon resonance is stronger when |*δ*_→_| is closer to zero, which is evident from Fig. 3. The exact positions of the peaks at ±5.3 MHz, explain the chosen detuning values for the second step at 776 nm, which were used to obtain Fig. 2: *δ*_↑_ = −2*π* × 3.5 MHz and *δ*_↓_ = 2*π* × 7.1 MHz. They are chosen such that the operational pathways are effectively equally detuned from the two-step two-photon resonance: *P*(→, ↓) is detuned by 2*π* × (−5.3 + 7.1) MHZ = 2*π* × 1.8 MHZ, and *P*(←, ↑) is detuned by 2*π* × (+5.3 − 3.5) MHZ = 2*π* × 1.8 MHZ.

## Discussion

**Figure 4:**
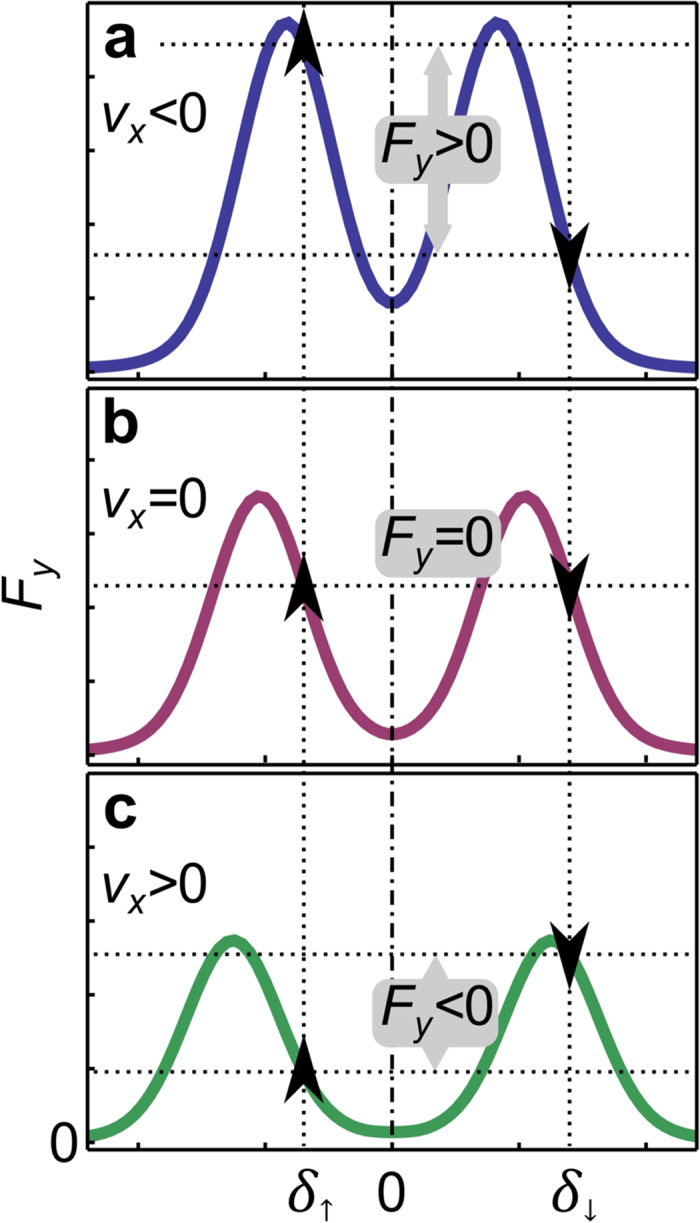
Interpretation of the synthetic Lorentz force from Figs 1 and 2, via two-step two-photon resonances presented in Fig. 3 Sketch of the resonant peaks that would be obtained in the setup shown in Fig. 3 for three atomic velocities: (**a**) *v*_*x*_ < 0, (**b**) *v*_*x*_ = 0, and (**c**) *v*_*x*_ > 0. Vertical dotted lines illustrate the values of the detuning used for the lasers aligned on the *y*-axis in Fig. 1. Arrows denote the direction of the force exerted by those lasers, and illustrate the way *F*_*y*_ observed in Fig. 2 arises as a function of velocity *v*_*x*_. See text for details.

Suppose that we repeat measurements corresponding to Fig. 3, but for an atomic cloud with mean velocity *v*_*x*_ different from zero. The results of such measurements would be identical as for *v*_*x*_ = 0, but the positions of the peaks would correspond to the Doppler shifted detuning values *δ*_→_ − *kv*_*x*_ and *δ*_←_ + *kv*_*x*_. Thus, because detuning can be mapped to velocity space, Fig. 3 can be reinterpreted as measurements for a fixed value of *δ*_←_ = −*δ*_→_, and for three different velocities *v*_*x*_ < 0 (4 MHz), *v*_*x*_ = 0 (6 MHz), and *v*_*x*_ > 0 (8 MHz). This is sketched in Fig. 4, where we see that the two peaks separate (approach) each other for *v*_*x*_ > 0 (*v*_*x*_ < 0, respectively).

We use Fig. 4 for a detailed explanation of the synthetic Lorentz force measured in Fig. 2. In measurements shown in Fig. 2, we have *δ*_↑_ < 0, which means that the positive force *F*_*y*_ > 0 in Fig. 2 results from the left resonance peak in Fig. 4. Likewise, because *δ*_↓_ > 0 was used for Fig. 2, the negative force *F*_*y*_ < 0 results from the right resonance peak in Fig. 4. The transverse force *F*_*y*_ measured in Fig. 2, can be approximately identified with 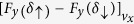, as illustrated in Fig. 4.

The choice of *δ*_↑_ and *δ*_↓_ in Fig. 2 is such that the strength of the forces arising from the two peaks balance each other, giving *F*_*y*_ = 0 for *v*_*x*_ = 0 [Fig. 4(b)]. Moreover, *δ*_↑_ and *δ*_↓_ are on the slopes of the two peaks in Fig. 4(b) for an atom with *v*_*x*_ = 0, where the maximal magnitude of Δ*F*_*y*_/Δ*v*_*x*_ is expected (detuning translates into velocity space via Doppler effect). For an atom with *v*_*x*_ < 0 [see Fig. 4(a)], the two peaks approach each other, yielding greater force from the left peak, which results in *F*_*y*_ > 0 for *v*_*x*_ < 0, and the opposite for *v*_*x*_ > 0 which yields *F*_*y*_ < 0 as shown in Fig. 4(c).

It should be pointed out that our scheme is inherently asymmetric. The intensity of the resonance peaks shown in Fig. 4 decreases when they separate (for *v*_*x*_ > 0), in contrast to when they approach each other (for *v*_*x*_ < 0). Thus, the net force along *y* is larger for negative *v*_*x*_, than for the velocity of the same magnitude but with a positive sign. By using a scheme with four atomic levels^17^, one could remedy the asymmetry in the force, present in the three-level scheme. In addition, the initial density of the atomic cloud is not identical prior to acceleration in the positive/negative direction due to the slight asymmetry of the displaced trapping potential. This also affects the transverse force due to absorption and multiple scattering^21^. The inherent asymmetry in the force *F*_*y*_(*v*_*x*_) and in the initial density of the cloud, is reflected in the asymmetry of the observed motion in Fig. 2 for *v*_*x*_ < 0 and *v*_*x*_ > 0. Importantly, all of these details only quantitatively affect the results, but not qualitatively.

## Conclusion

The presented experiment, which demonstrates the synthetic Lorentz force by using radiation pressure, is performed in a classical cold atomic gas, prepared in a MOT, where the heating due to spontaneous emission does not prevent the observations. The concept of a force is natural in our classical laser cooled system^18^. We would like to emphasize that our method is entirely different from the Berry phase approach, where the connection with the Lorentz force can be made in a semiclassical approximation^22^.

In the outlook, we foresee many intriguing novel experiments based on the presented method. First, one could develop experiments using more sophisticated schemes, involving more atomic levels (see Ref. [17]), to create a uniform synthetic magnetic field. Next, we plan to adjust our system for the observation of the predicted rotation of the cloud during expansion^17^. One of the goals of this research is to build up a toroidal trap for cold atoms with a toroidal synthetic magnetic field, which holds potential to emulate the plasma in a tokamak. The proposed concept could be used for velocity selection of atomic beams, or for developing a novel kind of mass spectrometer for neutral atoms. We believe that our concept or an analogous scheme could be applicable in other systems, such as suspended nanoparticles.

## Methods

### Experimental setup

The ^87^Rb MOT is set up in the standard *σ*^+^ *σ*^−^ retro-reflected configuration, with beam diameters of 2 cm. The trap is vapour loaded in a glass cell which facilitates fast switching of the magnetic field. Cooling and repumper lasers are external cavity diode lasers (ECDL) delivering total powers of 80 mW and 20 mW, respectively. The cooling laser is typically 2*π* × 24 MHz (4Γ) red-detuned from the 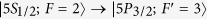 hyperfine transition. The repumper laser is in resonance with the 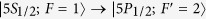 hyperfine transition, thus keeping most of population in the |5*S*_1/2_  ; *F* = 2〉 ground level. The quadrupole magnetic field gradient is 13 G/cm. The number of atoms in the trap is deduced by measuring the cloud fluorescence with a calibrated photodiode.

For the implementation of the synthetic Lorentz force two additional ECDL lasers were introduced in the experiment: one at 780 nm driving 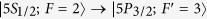 transition, and other at 776 nm driving 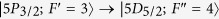 transition. Each laser beam is split into two beams which are sent on the rubidium cloud in counter-propagating configurations as shown in Fig. 1. Frequency and intensity control is done separately for each of the four laser beams with acoustic-optical modulators (AOM). After being frequency shifted, we couple the beams to single mode polarization maintaining fibres, which ensures linear polarization (in the *z*-direction) and uniform intensity.

All lasers used in the experiment are frequency stabilized by using modulation transfer spectroscopy. We modulate the laser diode current to modulate the laser frequency, which effectively increases the laser linewidth. This additional broadening is taken into account in theoretical calculations. The linewidths were checked by heterodyne beating of two stabilized lasers with similar locking parameters. For locking the laser tuned at 776 nm, we counter-propagate picked off beams from the 776 nm laser and the 780 nm laser through a heated ^87^Rb glass cell, where the 780 nm laser populates the 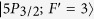 level. We observe an absorption signal resulting from the 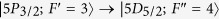 transition. This signal is mixed with the modulation signal from the 780 nm laser to obtain a frequency locking error signal. Therefore, there is no need to modulate the 776 nm laser frequency to stabilize it.

### Theoretical calculation of the radiation pressure force

To calculate the force plotted in Fig. 3, we first solve the optical Bloch equations (OBEs) to find the density matrix 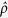
18:
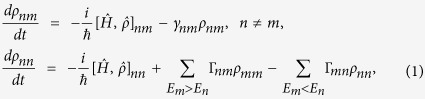
where *n*, *m* = 0, 1, 2. The Hamiltonian 
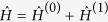
 describes a three-level system interacting with laser fields. 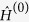 is represented by a diagonal matrix with elements 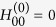, 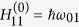, and 
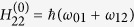
, whereas the interaction Hamiltonian is, in the dipole and the rotating wave approximation^18^,
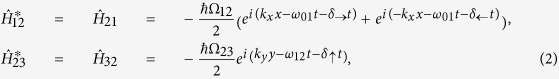
and zero otherwise. The linewidths, Γ_12_ = 2*π* × 6.1 MHz, Γ_23_ = 2*π* × 0.66 MHz, and the transition frequencies, *ω*_01_ = *k*_*x*_*c* and *ω*_12_ = *k*_*y*_*c*, where *k*_*x*_ = 2*π*/780 nm^−1^ and *k*_*y*_ = 2*π*/776 nm^−1^, correspond to the experiment; Γ_13_ = 0; the coherences are 
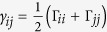
, where 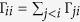. Because transition 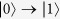 is driven with two lasers of different detuning values *δ*_←_ = −*δ*_→_, there is no stationary solution, and the level populations oscillate with the frequency |*δ*_→_| ~ 6 MHz (the phase of these oscillations depends on *x*, but this is not reflected in the force). The force on an atom is calculated by employing the Ehrenfest theorem^18^, and averaging over the oscillations: *F*′_*y*_(*δ*_→_, *δ*_←_, *δ*_↑_) 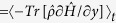. The average over time 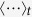 is justified because the oscillations at frequency |*δ*_→_| ~ 6 MHz are much faster than the characteristic scale for measuring the force, *τ*_*F*_, which is on the order of a few milliseconds.

The outlined procedure for calculating the force assumes that the lasers are perfectly monochromatic. However, there is finite laser linewidth that should be taken into account to quantitatively describe the two-step two-photon resonances in Fig. 3, i.e., *F*_*y*_(*δ*_↑_). The spectral profile of the diode laser can be described by a Gaussian in the frequency domain, 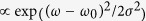. In the experiment, the modulation transfer spectroscopy for laser locking is used, and the central laser frequency is modulated with the amplitude *d* ≈ 2*π* × 1 MHz and frequency *η* = 2*π* × 14 kHz: ~w0 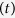 = *ω*_0_ + *d* sin *ηt*. Thus, the laser spectral profile, over time-scales larger than 1/*η*, is given by
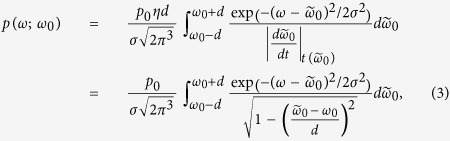
where *p*_0_ is the normalization factor.

The detuning values of 780 nm lasers oscillate as follows: 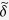
→ = *δ*_→_ + *d* sin*ηt* and 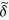 = *δ*_←_ + *d* sin*ηt*. Note that 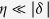
→, and at the same time 1/*η* is sufficiently smaller than *τ*_*F*_. This means that we can employ the separation of scales to take into account finite laser linewidth. We calculate the resulting force *F*_*y*_(*δ*_↑_) on the cloud by averaging 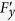 (*δ*_→_, *δ*_←_, *δ*_↑_) with the appropriate laser profile distribution in the frequency domain:
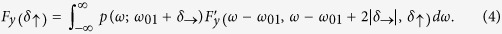


The width *σ* was determined to be 2*π* × 1.5 MHz by fitting to the experimental profiles. The Rabi frequencies used in the calculation are discussed in the Results Section.

## Additional Information

**How to cite this article**: Šantić, N. *et al*. Experimental Demonstration of a Synthetic Lorentz Force by Using Radiation Pressure. *Sci. Rep*. **5**, 13485; doi: 10.1038/srep13485 (2015).
